# Genotyping and antibiotic susceptibility of *Campylobacter* species isolated from raw milk samples in Qazvin, Iran

**DOI:** 10.1186/s13104-023-06576-9

**Published:** 2023-11-06

**Authors:** Zohreh Ahmadi, Babak Pakbin, Maryam kazemi, Zahra Rahimi, Razzagh Mahmoudi

**Affiliations:** 1 Department of Food Safety and Health, School of Public Health, Qazvin University of Medical Sciences, Qazvin, Iran; 2 Werner Siemens Chair of Synthetic Biotechnology, Dept. of Chemistry, Technical University of Munich (TUM), Lichtenberg Str. 4, 85748 Garching bei München, Germany; 3 Medical Microbiology Research Center, Qazvin University of Medical Sciences, Qazvin, Iran

**Keywords:** *Campylobacter *species, Antibiotic susceptibility, Genetic diversity, Raw milk

## Abstract

**Objective:**

*Campylobacter* species are major causes of foodborne illnesses, with unpasteurized milk being a significant carrier of these bacteria, posing a public health risk. One of the challenges in managing *Campylobacter* infections is the emergence and spread of antibiotic resistance. We conducted a study in Qazvin, Iran, testing 84 raw cow’s milk samples to determine the frequency of *C. jejuni* and *C. coli* using culture-based and multiplex PCR methods. Additionally, the disk diffusion and RAPD-PCR approaches were utilized to evaluate the phenotypic antibiotic resistance profile and genetic diversity of *Campylobacter* strains.

**Results:**

The findings indicated that *Campylobacter* spp. was present in 19.05% of the samples, with *C. coli* being the predominant isolate. We tested eight antibiotic agents, and the resistance levels of the isolates were as follows: erythromycin 100%, tetracycline 75%, doxycycline 56.25%, ceftriaxone 43.75%, chloramphenicol 37.5%, amoxicillin-clavulanic acid 25%, nalidixic acid 12.5%, and azithromycin 6.25%. Genetic diversity analysis categorized *Campylobacter* isolates into 39 clusters, indicating a wide diversity among strains. However, no significant correlation was observed between antibiotic resistance and cluster patterns. These findings underscore the role of raw milk as a reservoir for *Campylobacter* spp. and highlight the substantial antibiotic resistance and genetic diversity within the species population.

## Introduction

*Campylobacter* spp. are widely recognized as prevalent enteric pathogens, significantly contributing to bacterial gastroenteritis in humans worldwide. These Gram-negative bacteria are responsible for over 500 million infections, which can be fatal for vulnerable individuals, such as children or the elderly [1]. As zoonotic foodborne pathogens, they can colonize the intestinal tracts of animals and are primarily transmitted to humans through direct contact or the consumption of contaminated animal-derived food, such as poultry and raw milk [2]. The two main strains responsible for contamination and illness are *C. jejuni* and *C. coli.* [3]. *Campylobacter* infection can manifest with various symptoms, including diarrhea, abdominal pain, fever, nausea, and, in severe cases, prolonged digestive problems. While *Campylobacter* typically induces self-limiting symptoms, it may necessitate antimicrobial treatment in severe cases [3, 4]. Antibiotics are frequently employed for disease prevention and treatment, but their overuse leads to the emergence of antimicrobial resistant (AMR) bacteria. An alarming trend is the growing resistance observed in *Campylobacter* spp. to multiple antibiotics, including macrolides, aminoglycosides, quinolones, and tetracyclines [5, 6]. These microorganisms lead to a yearly increase in mortality and result in significant economic costs [7]. Moreover, the existence of antibiotics and their metabolic residues in the environment profoundly impacts the structure and diversity of microbial populations [8]. Understanding the genetic diversity and population structure of *Campylobacter* can aid epidemiological investigations, outbreak management, and the development of effective control strategies. RAPD-PCR is widely used for molecular genotyping, revealing genetic variability through distinct banding patterns from random bacterial genome regions, which provides valuable insights into the genetic variability within populations of organisms [9].

In recent years, the supply and distribution of raw milk in Iran has increased. Nevertheless, limited studies have examined the associated risks of its consumption. Therefore, this study aims to enhance our understanding of *Campylobacter* spp. prevalence, explore antibiotic resistance, and genetic variations in isolates from raw milk samples collected in Qazvin, Iran.

## Materials and methods

### Collection of samples

In July 2021, a total of 84 random samples of unpasteurized cow’s milk were purchased from retail markets located in three areas of Qazvin Province, Iran [10]. All samples were collected under strict sanitary conditions, and placed in an insulated icebox at 4 °C until delivery to the laboratory of food microbiology at Qazvin University of Medical Science for further analysis.

### Isolation of *Campylobacter* spp.

Twenty-five milliliters of each milk sample was centrifuged at 20,000 × g for 35 min at 8 °C. After discarding the supernatant, the pellet was mixed with 45 mL of Bolton broth containing *Campylobacter-*selective supplement (HiMedia, India) and 5% defibrinated sheep blood (Baharafshan, Iran), and then incubated for 48 h at 42 °C under microaerophilic conditions (10% CO2, 85% N2 and 5% O2) using a Gas Pack C (Merck, Germany). Following incubation, a 30 µL aliquot of enriched cultures was streaked onto mCCDA (QUELAB,Canada) with antibiotic *Campylobacter*-selective supplement (Oxoid, UK) and incubated as previously mentioned. The suspected *Campylobacter* colonies were subjected to examination of morphology, oxidase, and catalase activity [11, 12]. Subsequently, these colonies were preserved in BHI broth with glycerol, and kept at − 80 °C for further investigation.

### Identification of *Campylobacter* spp.

A boiling procedure was employed for the extraction of DNA from isolates. A multiplex PCR technique targeting the 16 S rRNA gene was employed for molecular identification. Specifically, the primer pair 0301 (F, CTT AAA GCN ATG ATA GTR GAY AAR) and 0304 (R, ACA GGR ATT CCR CGY TTT GTY TC), was used to target all *Campylobacter* species, and the isolates were differentiated as *C.jejuni* and *C.coli* using specific IpxA genes [13]. The PCR mixture reaction (20 µL) consisted of 10 µL Master Mix (Ampliqon, Denmark), 2 µL of each primer, 5 µL DNA template, and 1 µL deionized water. The PCR proceeded as follows: initial denaturation at 95 °C for 5 min, followed by 45 cycles of denaturation at 95 °C for 40 s, annealing at 55 °C for 40 s, and extension at 72 °C for 1 min. The final extension phase lasted 7 min at 72 °C. Subsequently, the PCR products underwent electrophoresis on a 1.5% *w/v* agarose gel in 0/5× TBE buffer with DNA-safe stain (CinnaGen, Iran), running at 110 V for 75 min. The results were photographed using a gel documentation system (NovinPars Co., Iran) [14]. *C. jejuni* ATCC 33,291 and *C. coli* ATCC 43,478 were used as control strains.

### Genotyping by RAPD-PCR

In the RAPD genotyping analysis of *Campylobacter* isolates, a primer with the sequence 5′-CGCGTGCCAG-3′ was employed. A 25 µl reaction volume was prepared, consisting of 2 µl of DNA template (50 ng/µl), 12.5 µl of PCR master mix, 1 µl of primer (0.2 M/µl), and deionized sterile water was used for RAPD amplification. The thermal cycling program proceeded as follows: 95 °C for 5 min, 1 min at 36 °C, 4 min at 72 °C, and then 35 cycles of 95 °C for 1 min, 36 °C for 1 min, with a final extension at 72 °C for 4 min. Amplified RAPD-PCR products were electrophoresed on a 1.5% *w/v* agarose gel containing 0.5X TBE buffer with staining, running at 100 V for 1 h. The gels were visualized using a Gel Doc system. All analyses of UPGMA dendrograms were performed using PyElph and NTsys software [15].

### Antibiotic susceptibility testing

In this study, eight antibiotic disks (Padtan teb) were used, including tetracycline (30 g), erythromycin (15 g), doxycycline (30 g), azithromycin (15 g), nalidixic acid (30 g), chloramphenicol (30 g), ceftriaxone (30 g), and amoxicillin/clavulanic acid (30 g) [16]. The disc diffusion technique was conducted using the Kirby-Bauer method on Mueller-Hinton agar, following CLSI guidelines [17]. CLSI *Enterobacteriaceae* breakpoints were used to interpret resistance.

#### Statistical analysis

We employed the Chi-squared test and Fisher’s exact test to assess significant differences (*P* value ≤ 0.05) among the incidence rates. We conducted these analyses using SPSS version 22.0.1 (SPSS, Chicago, IL, USA).

## Results

Our study yielded significant findings regarding *Campylobacter* contamination in the tested samples. *Campylobacter* was found in 16 samples (19.05%, n = 84), with 10 (62.5%) identified as *C. coli* and 6 (37.5%) as *C. jejuni*. The highest contamination rates were observed in the southern regions of the city (24%) (Table 1).

**Table 1:** Prevalence of *Campylobacter* isolates from different sources in Qazvin City, Iran

Examined samples (Raw Milk)	No.of samples	No.of *Campylobacter* *Spp.*	(%)	No.of *Campylobacter* *Jejuni*	(%)	No.of *Campylobacter* *Coli*	(%)
North	26	3	11.54%	1	33.33%	2	66.67%
Center	33	7	21.21%	4	57.15%	3	42.86%
South	25	6	24%	1	16.67%	5	83.33%
Total	84	16	19.05%	6	37.5%	10	62.5%

Furthermore, all sixteen *Campylobacter* isolates were assessed for antimicrobial resistance against eight antibiotic agents, and the results are presented in Table 2. A striking observation was that all isolates exhibited resistance to erythromycin. Tetracycline resistance was also notably prevalent compared to other antibiotics. Both *C. jejuni* and *C. coli* strains demonstrated considerable resistance to doxycycline and ceftriaxone, while the lowest resistance rates among *Campylobacter* isolates were observed against azithromycin.

**Table 2:** Antibiotic resistance phenotype of *C. jejuni* and *C. coli* isolated from the raw milk samples

		n (%)		
Antibiotic Class	Antibiotic Agent	*C. jejuni* (n = 6)	*C. coli* (n = 10)	Total (n = 16)
β-lactams	Amoxicillin-clavulanic acid	2(33.34)	2(20)	4(25)
	Ceftriaxone	2(33.34)	5(50)	7(43.75)
Quinolone	Nalidixic acid	0(0)	2(20)	2(12.5)
Phenicol	Chloramphenicol	3(50)	3(30)	6(37.5)
Tetracyclines	Tetracycline	6(100)	6(60)	12(75)
	Doxycycline	4(66.67)	5(50)	9(56.25)
Macrolides	Erythromycin	6(100)	10(100)	16(100)
	Azithromycin	1(16.67)	0(0)	1(6.25)

**Fig. 1:**
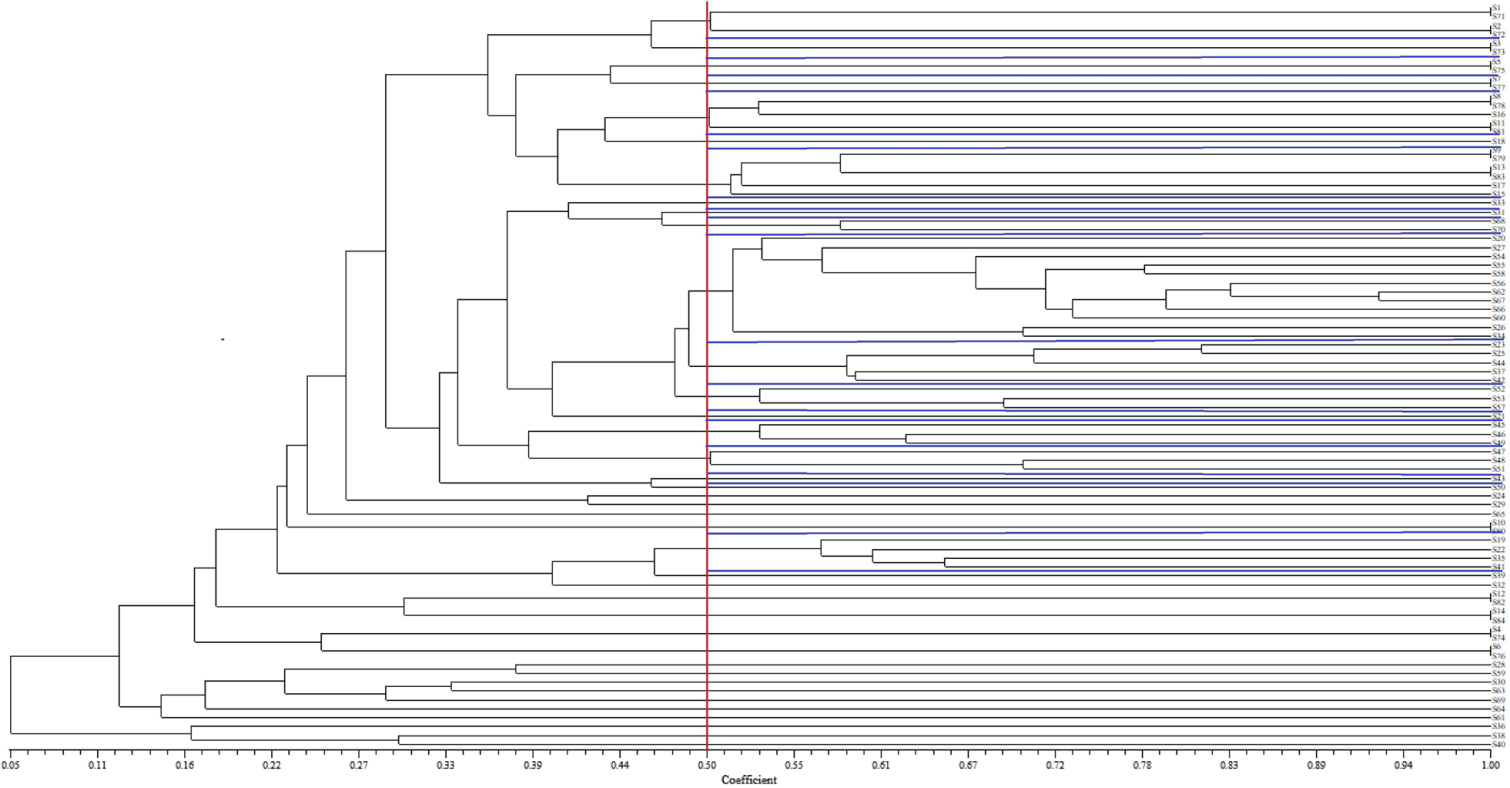
Phylogenetic tree of Campylobacter strains isolated from raw milk samples (with at least a 50% similarity coefficient)

The UBC245 arbitrary primer in RAPD-PCR amplified *Campylobacter* isolates, producing diverse patterns with three to ten bands ranging from 200 to > 2200 bp. This primer effectively differentiated the *Campylobacter* isolates from the eighty-four milk samples into 39 distinct clusters (R1-R39), with at least a 50% similarity coefficient (Fig. 1). Simpson’s index of diversity, calculated as 0.93, indicated a high genetic diversity among the investigated isolates. Genotypic diversity in these bacteria can signify their ability to thrive and evolve within a reservoir. However, there was no significant relationship observed between antibiotic resistance and genotyping patterns.

## Discussion

Milk has been identified as a common vehicle for *Campylobacter* contamination, with several outbreaks in different countries, such as the Netherlands [18], the USA [19, 20], Sweden [21], Denmark [22], and England [23], highlighting the dangers associated with the consumption of unpasteurized dairy and the subsequent rise in *Campylobacter* infections. In this study, we aimed to determine *Campylobacter* prevalence in milk within different regions of Qazvin, Iran. The prevalence rate of *Campylobacter* spp. was observed at 19.05%, which was higher than rates reported in Pakistan (10.2%) [24], Erbil, Iraq (12.6%) [25], Northern Italy (12%) [26], Sweden (9%) [27], and Poland (11.8%) [28]. In contrast, the frequencies observed here were lower than those reported from Tanzania (35.4%) [29], and Egypt (82.98%) [3]. On the other hand, a previous study in Iran by Haghi et al. reported the absence of *Campylobacter* in milk samples obtained from dairy bovines, suggesting that infections typically arise from secondary contamination [30]. In general, the *Campylobacter* prevalence variations in findings among studies make it challenging to establish a direct link. Influential factors such as farm location, climate, seasonal elements, and husbandry systems may contribute to these differences [31, 32].

The majority of *Campylobacter* infections are primarily caused by *C. jejuni*, which is commonly found in milk samples [10]. In our study, we identified *C. coli* as the dominant strain in isolates, with a detection frequency of 65.5%. This finding is consistent with some reports [33, 34]. Conversely, more studies, such as those conducted by Kabir et al. [35], Andrzejewska et al. [28] and Raeisi et al. [36], have reported *C. jejuni* as the prevailing strain. Based on Kalantar et al. ‘s research [37], one possible reason for the rising prevalence of *C. coli* strains, may be linked to the repetitive administration of antimicrobial treatments in specific areas and its selective impact on a particular population, which can lead to subsequent resistance development in this particular species.

The proliferation of AMR bacteria is a serious global concern. This issue is particularly significant in some parts of Asia, as indicated by the findings of the WHO [38]. In Iran, widespread antimicrobial usage, self-medication practices, limited public knowledge, and a lack of veterinary legislation have contributed to the rise of multidrug resistant (MDR) strains, posing significant challenges to the healthcare system [39]. In this study, all the isolates displayed significant resistance to erythromycin, a first-generation macrolide antibiotic. This finding is consistent with previous research in Egypt by Naeni et al., which reported 100% resistance to erythromycin in *Campylobacter* isolates from various sources [3]. The high prevalence of erythromycin resistance is concerning, as it is commonly used for treating human *Campylobacter* infections [6]. Furthermore, tetracycline, doxycycline, and chloramphenicol are often considered alternative therapies for diarrhea patients [2]. Our examination revealed a significant level of resistance to both tetracycline and doxycycline. This result aligns with a report by Igwaran and Okoh (2020), who found a high rate of phenotypic resistance to tetracycline and doxycycline in *Campylobacter* isolates (83.33% and 87.65%, respectively) [34]. Nalidixic acid testing showed 12.5% resistance, lower than in prior studies [12, 40]. This could be attributed to limited exposure of this antibiotic to farm environments. Furthermore, when assessing β-lactam antibiotics, isolates demonstrated notable resistance. These findings underscore the diverse applications of the β-lactam family in veterinary medicine [41]. Our findings indicate that all isolates were resistant to multiple antimicrobial agents. The excessive use of antibiotics in farming contaminates the environment, fostering resistance in animals and facilitating gene transfer. Addressing this issue requires implementing strategies such as rational antibiotic use, improved infection control, and the development of new antibiotics to safeguard the food chain and the well-being of both humans and animals.

We observed a high level of genetic diversity among strains isolated from raw milk samples, resulting in 39 distinct clusters within the *Campylobacter* isolates. These results are in agreement with those reported by Chuma et al. [9]. Horizontal gene transfer and genomic reconfiguration are potential mechanisms contributing to the genetic diversity observed among *Campylobacter* strains. Similar patterns of diversity have been observed in other *Enterobacteriaceae* family strains, such as *Shigella*, *E. coli*, and *Salmonella*, in previous studies of food samples. This substantial diversity is likely influenced by various sources of contamination. These sources include the transfer of *Campylobacter* strains from farm animals and food production environments to humans. Additionally, it may arise from the consumption of foods contaminated with various foodborne pathogens and international travel.

## Conclusions

This study showed a high incidence of *Campylobacter* isolation, predominantly *C. coli*, emphasizing the risks of consuming raw milk. Additionally, significant antibiotic resistance and genetic variations were found among *Campylobacter* spp. The data presented in this study are alarming and magnify the need for monitoring, detecting, and controlling the spread of foodborne bacteria, particularly MDR species.

### Limitations

To assess the genetic relatedness and antimicrobial susceptibility, 16 *Campylobacter* isolates from milk samples are not sufficient, and inadequate funding has been a limiting factor for this research project.

## Data Availability

We confirm that all data included in this study are available within the article.

## Abbreviations

RAPD-PCR: Rapid amplification of polymorphic DNA-polymerase chain reaction
mCCDA: Modified cefoperozone charcoal deoxycholate agar
BHI: Brain heart infusion
ATCC: American Type Culture Collection
CLSI: Clinical and Laboratory Standards Institute
WHO: World Health Organization
E. coli: Escherichia coli

## References

[1] Campylobacter. 2020. https://www.who.int/news-room/fact-sheets/detail/campylobacter.

[2] Igwaran A, Okoh AI (2019). Human campylobacteriosis: a public health concern of global importance. Heliyon..

[3] El-Naenaeey E-s, El-Hamid MA, Khalifa E (2021). Prevalence and antibiotic resistanc patterns of *Campylobacter* species isolated from different sources in Eygpt. J Microbiol Biotechnol food Sci.

[4] Ammar AM (2021). Campylobacter as a major foodborne pathogen: a review of its characteristics, pathogenesis, antimicrobial resistance and control. J Microbiol Biotechnol food Sci.

[5] Qin X, Wang X, Shen Z (2023). The rise of antibiotic resistance in *Campylobacter*. Curr Opin Gastroenterol.

[6] Dai L (2020). New and alternative strategies for the prevention, control, and treatment of antibiotic-resistant *Campylobacter*. Transl Res.

[7] Ma F (2021). Use of antimicrobials in food animals and impact of transmission of antimicrobial resistance on humans. Biosaf Health.

[8] Zhang Q, Beyi AF, Yin Y (2023). Zoonotic and antibiotic-resistant Campylobacter: a view through the One Health lens. One Health Adv.

[9] Chuma IS (2016). Epidemiology and RAPD-PCR typing of thermophilic campylobacters from children under five years and chickens in Morogoro Municipality, Tanzania. BMC Infect Dis.

[10] Admasie A et al. Seasonal variation in the prevalence and antimicrobial resistance of *Campylobacter* in milk and milk products in Ethiopia. SSRN 4479841.10.2139/ssrn.4479841.

[11] Del Collo LP (2017). Prevalence, antimicrobial resistance, and molecular characterization of *Campylobacter* spp. in bulk tank milk and milk filters from US dairies. J Dairy Sci.

[12] Modi S (2015). Prevalence of *Campylobacter* species in milk and milk products, their virulence gene profile and anti-bio gram. Vet World.

[13] Klena JD (2004). Differentiation of *Campylobacter coli*, *Campylobacter jejuni*, *Campylobacter lari*, and *Campylobacter upsaliensis* by a multiplex PCR developed from the nucleotide sequence of the lipid A gene lpxA. J Clin Microbiol.

[14] Wang G (2002). Colony multiplex PCR assay for identification and differentiation of *Campylobacter jejuni*, *C. Coli*, *C. lari*, *C. Upsaliensis*, and *C. fetus* subsp. fetus. J Clin Microbiol.

[15] Carvalho AC (2001). Molecular characterization of invasive and noninvasive *Campylobacter jejuni* and *Campylobacter coli* isolates. J Clin Microbiol.

[16] Alaboudi AR (2020). Prevalence, antibiotic resistance and genotypes of *Campylobacter jejuni* and *Campylobacter coli* isolated from chickens in Irbid governorate, Jordan. Int J Food Microbiol.

[17] Weinstein MP. Performance standards for antimicrobial susceptibility testing. Clinical and Laboratory Standards Institute; 2021.

[18] Heuvelink AE (2009). Two outbreaks of campylobacteriosis associated with the consumption of raw cows’ milk. Int J Food Microbiol.

[19] Davis KR (2016). *Campylobacter jejuni* infections associated with raw milk consumption—Utah, 2014. Morb Mortal Wkly Rep.

[20] Burakoff A (2018). Outbreak of fluoroquinolone-resistant *Campylobacter jejuni* infections associated with raw milk consumption from a herdshare dairy—Colorado, 2016. Morb Mortal Wkly Rep.

[21] Lahti E (2017). Outbreak of campylobacteriosis following a dairy farm visit: confirmation by genotyping. Foodborne Pathog Dis.

[22] Institut SS. Campylobacter infections, 2016–2017. Annual reports on disease incidence, Denmark 2018. 2018. https://en.ssi.dk/surveillance-and-preparedness/surveillance-in-denmark/annual-reports-on-disease-incidence/campylobacter-infections-2016-2017.

[23] Kenyon J (2020). Campylobacter outbreak associated with raw drinking milk, North West England, 2016. Epidemiol Infect..

[24] Hussain I (2007). Prevalence of *Campylobacter* species in meat, milk and other food commodities in Pakistan. Food Microbiol.

[25] Almashhadany DA. Isolation, biotyping and antimicrobial susceptibility of *Campylobacter* isolates from raw milk in Erbil city, Iraq. Italian J Food Saf. 2021. 10.4081/ijfs.2021.8589. 10.4081/ijfs.2021.8589 PMC7970396 33747984

[26] Bianchini V (2014). Prevalence in bulk tank milk and epidemiology of *Campylobacter jejuni* in dairy herds in Northern Italy. Appl Environ Microbiol.

[27] Artursson K (2018). Foodborne pathogens in unpasteurized milk in Sweden. Int J Food Microbiol.

[28] Andrzejewska M (2019). Prevalence, virulence, and antimicrobial resistance of *Campylobacter* spp. in raw milk, beef, and pork meat in Northern Poland. Foods.

[29] Kashoma IP (2015). Antimicrobial resistance and genotypic diversity of Campylobacter isolated from pigs, dairy, and beef cattle in Tanzania. Front Microbiol.

[30] Haghi F (2015). Detection of major food-borne pathogens in raw milk samples from dairy bovine and ovine herds in Iran. Small Rumin Res.

[31] Barata AR (2022). Occurrence and seasonality of *Campylobacter* spp. in Portuguese dairy farms. Int J Food Microbiol.

[32] Bertasi B, et al. Seasonal variability of thermophilic *Campylobacter* spp. in raw milk sold by automatic vending machines in Lombardy Region. Italian J Food Saf. 2016;5(3). 10.4081/ijfs.2016.5848. 10.4081/ijfs.2016.5848 PMC5090118 27853714

[33] Sanad YM (2011). Genotypic and phenotypic properties of cattle-associated Campylobacter and their implications to public health in the USA. PLoS ONE..

[34] Igwaran A, Okoh AI (2020). Occurrence, virulence and antimicrobial resistance-associated markers in *Campylobacter* species isolated from retail fresh milk and water samples in two district municipalities in the Eastern Cape Province, South Africa. Antibiotics.

[35] Kabir SL (2018). Isolation, molecular identification and antimicrobial resistance patterns of *Campylobacter* species of dairy origin: first report from Bangladesh. Vet Sci Dev..

[36] Raeisi M (2017). Antimicrobial resistance and virulence-associated genes of *Campylobacter* spp. isolated from raw milk, fish, poultry, and red meat. Microb Drug Resist.

[37] Kalantar M (2017). Monitoring the virulence genes in *Campylobacter coli* strains isolated from chicken meat in Tehran, Iran. Infect Epidemiol Microbiol.

[38] Organization WH. Critically important antimicrobials for human medicine; 6th revision; Geneva, Switzerland. 2022. https://www.who.int/publications/i/item/9789241515528.

[39] Sharif Z (2021). Irrational use of antibiotics in Iran from the perspective of complex adaptive systems: redefining the challenge. BMC Public Health.

[40] Kashoma IP (2016). Prevalence and antimicrobial resistance of Campylobacter isolated from dressed beef carcasses and raw milk in Tanzania. Microb Drug Resist.

[41] Rana EA, Fazal MA, Alim MA (2022). Frequently used therapeutic antimicrobials and their resistance patterns on *Staphylococcus aureus* and *Escherichia coli* in mastitis affected lactating cows. Int J Vet Sci Med.

